# Fingerprints of a message: integrating positional information on the transcriptome

**DOI:** 10.3389/fcell.2014.00039

**Published:** 2014-08-14

**Authors:** Erik Dassi, Alessandro Quattrone

**Affiliations:** Laboratory of Translational Genomics, Centre for Integrative Biology, University of TrentoTrento, Italy

**Keywords:** transcriptome, integration, post-transcriptional control, translation, RNA-seq, mRNA, data format, standards

## Abstract

The recent explosion of high-throughput sequencing methods applied to RNA molecules is allowing us to go beyond the description of sequence variants and their relative abundances, as measured by RNA-seq. We can now probe for RNA engagement in polysomes, for ribosomes, RNA binding proteins and microRNAs binding sites, for RNA secondary structure and for RNA methylation. These descriptors produce a steadily growing multidimensional array of positional information on RNA sequences, whose effective integration only would bring to decipher the regulatory interplay occurring between proteins, RNAs and their modifications on the transcriptome. This interplay ultimately dictates the degree of mRNA availability to translation, and thus the occurrence of cell phenotypes. However, several issues in data presentation are slowing down effective integration. A standardization effort for new dataset types produced should be urgently undertaken to solve these issues. Providing uniformed experimental details along with datasets processed to be directly usable and employing shared formats would greatly simplify integration efforts, strengthening hypotheses stemming from correlative observations and eventually bringing to mechanistic understanding.

## Probing the biological status of whole transcriptomes

The last 15 years have witnessed, starting with the advent of microarray-based gene expression probing, an explosion of high-throughput technologies for the characterization of biological molecules. These technologies, affordable and relatively simple to apply, are steadily paving the way for routine multi-omics studies. The latest of such technologies, high-throughput sequencing (HTS) (Metzker, 2010), has quickly gained widespread acceptance and concurrently enabled several different types of measurements. Its sequence-based nature, permitting to pinpoint relevant features on the genome or transcriptome of interest (position-aware data), and its massively parallel data production capabilities are now indeed applied to the study of a wide array of biological questions. Applications focus on DNA (identification of sequence and copy number variants, mapping of chromatin binding sites by transcription factors and other proteins, chromatin topology studies in nuclei, etc.) (Koboldt et al., 2013) and on RNA (sequence variants of mRNAs and non-coding RNAs, expression levels, mapping of binding sites of RNA binding proteins (RBPs), post-transcriptional modifications etc.), (Ascano et al., 2013; Mutz et al., 2013). Translational regulation of gene expression, in particular, has lately been object of increasing interest: its role in profoundly reshaping transcriptome variations and being the determinant of plasticity in the nascent proteome (Vogel et al., 2010; Stevens and Brown, 2013) is increasingly appreciated. Consequently, omic approaches have been developed to investigate which features of an mRNA may influence its translation rate, which trans-factors play a role in such regulatory processes and how these two aspects combine to yield the final protein levels. We will focus on RNA-centered methods to examine the types of biological information they can provide; we will then look at how this information should be integrated to allow us a better understanding of both the global transcriptome dynamics and their effects on phenotype.

**Figure 1:**
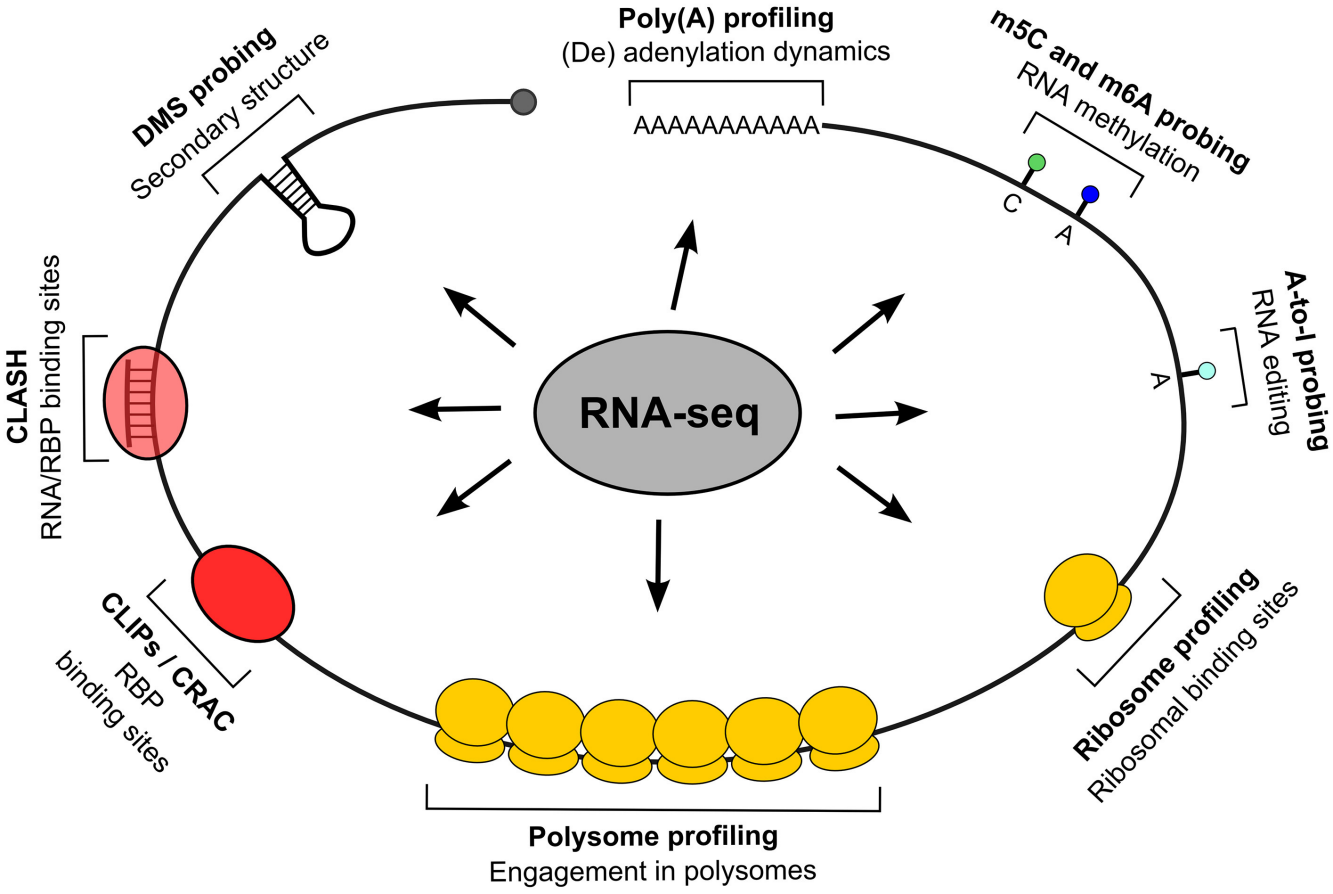
**Techniques for positional whole-transcriptome probing**. The figure displays techniques allowing to study transcriptomes at various observational levels, with particular regard to positional information; all techniques, indicated by their representative feature on transcripts, are based on RNA-seq.

As shown in Figure 1, such methods can be classified by their descriptive capability, either *molecular* for the entire RNA or *submolecular* for specific RNA portions, and the kind of description they provide, *quantitative*, *qualitative* or both. The description of entire transcripts is provided by RNA-seq (Mutz et al., 2013), an HTS-based method which gives the sequence of coding and non-coding transcripts, including mapping of alternative transcription or termination sites, splice variants produced on the same locus and the presence of expressed sequence polymorphisms. Since different transcripts can be quantified in their relative abundance, this type of information is both qualitative and quantitative. The polysome profiling method (Arava, 2003; Gandin et al., 2014) is based on the separation by sucrose gradient centrifugation of cellular fractions containing polysomes and the subsequent quantification of their mRNA relative (to the total lysate or to the fractions not containing polysomes) abundance, which can be performed by RNA-seq or by the more conventional microarray analysis. The resulting information is a quantitative and qualitative description of the degree of polysomal engagement for every transcript (by which the molecular nature of this method), the so called translatome (Tebaldi et al., 2012); a calculation of translational efficiency can be done by this assay. The qualitative component of polysome profiling is given by computational approaches which allow us to investigate the differential association of mRNAs produced by the same gene locus (splice and 5′/3′ variants) with the polysomes (Frac-seq, Sterne-Weiler et al., 2013), or which measure the effect of single-nucleotide polymorphisms on translational efficiency (Li et al., 2013). Ribosome profiling (Ingolia, 2014) aims at providing a snapshot of mRNAs under translation by scoring the transcript regions which are protected from nuclease attack by ribosomes. It is a RNA-seq-based method of the submolecular type: obtainable information can be integrated at the transcript level but has a positional content, so that translation initiation and termination sites, potential translation stalling events, upstream ORF translation, can be derived (Ingolia et al., 2011). Besides engagement in translation, another type of general, qualitative description of transcript status is the secondary structures pattern, recently become available to profiling through nucleotide accessibility probing coupled with RNA-seq (Ding et al., 2014; Rouskin et al., 2014; Talkish et al., 2014; Wan et al., 2014). Eventually, a transcript component which can be investigated is the poly(A) tail: two recent methods, PAL-seq (Subtelny et al., 2014) and TAIL-seq (Chang et al., 2014), exploit RNA-seq to characterize its length and potential modifications (such as uridylation and guanylation). The same principle of nuclease protection exploited in ribosome profiling is then systematically applied in locating RNA-associated “footprints” of RBPs. The CLIP techniques family: HITS-CLIP, PAR-CLIP, and iCLIP (Ule et al., 2003; Hafner et al., 2010; Konig et al., 2010) and the CRAC approach (Granneman et al., 2009) exploit an UV-induced crosslinking of RNA and associated proteins (with the option of using photoactivatable nucleotides, as done in PAR-CLIP) to enable the identification of RNA targets and binding sites for single, immunoprecipitated RBPs. These are therefore submolecular and essentially qualitative approaches. A variant method, CLASH (Helwak et al., 2013), introduces a RNA ligation step to locate sites where other RNAs are associated in trans in a protein complex, allowing to experimentally identify miRNA binding sites. CLIP methods can also be extended to consider many RBPs at once: “global CLIP” approaches such as protein occupancy profiling (Baltz et al., 2012) and PIP-seq (Silverman et al., 2014) thus provide contact sites for all RBPs at once on a transcriptome.

Coming finally to the most submolecular level, that of single nucleotides, mRNA editing events (such as adenosine to inosine conversions) can be revealed either by inosine chemical erasing (ICE), as in Sakurai et al. (2014), or by directly looking for sequence variants in RNA-seq reads (St. Laurent et al., 2013; Bazak et al., 2014). Eventually, RNA 5-methylcytosine and N6-methyladenosine nucleotide methylation can be detected with single-nucleotide precision, respectively by bisulfite conversion (Squires et al., 2012; Edelheit et al., 2013) and immunoprecipitation (Dominissini et al., 2012; Meyer et al., 2012; Khoddami and Cairns, 2013) or by other biochemical methods (Hussain et al., 2013; Liu et al., 2013).

## Approaches for the integration of transcript-centered omics

Currently, several hundred papers employing the described transcriptome-based omics methods have been published, including a considerable number of pure RNA-seq datasets, secondary structure probing, editing and methylation profiles for the most common cell lines and organisms (see Figure 1), and at least 40 different CLIP or CLIP-like datasets (Dassi et al., 2014). With such a huge amount of data available, the naturally arising question is how to integrate these different types of information to obtain more insights than if considering single datasets in isolation. Several works have approached this problem so far. As shown in Table 1, they can be classified according to the different perspectives adopted in doing so.

**Table 1:** **Current approaches for positional information integration on the transcriptome**.

**Name**	**Description**	**Scope**	**Potential issues**	**References**
Integrated databases	Collecting and presenting available datasets of heterogeneous types and biological sources; allowing users to mine the data types in combination	Global over a vast number of different data types	Data quality and processing assessment not always possible; achieving database completeness and constant content update is particularly time-intensive	Anders et al., 2012; Dassi et al., 2012, 2014; Li et al., 2014
Multi-level profiling	Performing various types of measurements (i.e., mRNA levels, RNA secondary structure, RNA methylation) in the same system of interest (e.g., cell line) to derive correlative patterns	Global over a limited number of data types	Need very different experimental and data analysis expertise; results applicability is limited to the studied system	Genolet et al., 2011; Bates et al., 2012; Clarke et al., 2012; Dominissini et al., 2012; Fu et al., 2012; Tebaldi et al., 2012; Courtes et al., 2013; Dudek et al., 2013; Willimott et al., 2013; Zheng et al., 2013; Ding et al., 2014; Mao et al., 2014; Wang et al., 2014a
Measurements & public data exploitation	Performing a small number of measurements (i.e., mRNA levels only) in the system of interest, and exploiting public data to study genes derived from these measurements (i.e., presence of translational regulation) to infer and validate potential regulatory mechanisms and patterns	Over a small number (dozens) of interesting genes	Publicly available data on the system one wants to use may not be available; further validation and/or mechanistic experiments may be needed	Mazza et al., 2013; Avery-Kiejda et al., 2014; Schueler et al., 2014; Wang et al., 2014b

The table describes currently applied approaches to the integration of position-aware RNA datasets. Scope of the various approaches and associated potential issues are outlined along with the references of works employing them.

A first, post-experimental way of integrating these heterogeneous data sets consists in building a database presenting all the collected data together, thus allowing users to prioritize and validate potential connections. Mining the data, superimposed on a reference genome, can be approached by looking for single genes (as happens in genome browsers) or by studying interesting gene lists (e.g., through functional enrichment or co-regulation analyses). This road was taken by AURA/AURA2 (Dassi et al., 2012, 2014), DORiNA (Anders et al., 2012), and starBase (Li et al., 2014). The first provides RBP and miRNA binding sites, cis-elements sites, RNA editing, and methylated nucleotides; the second offers RBP binding sites and predicted miRNA targets; the last includes RBP binding sites and miRNA interactions with coding and non-coding RNAs. While these databases are of general interest and can be useful for a broad spectrum of preliminary investigations, they still mostly contain data obtained in a limited set of particularly common model systems or cell lines (e.g., HEK293 cells): users will then likely need to trust this information to hold in their system of interest or validate the interaction in their specific conditions (e.g., for an RBP-mRNA interaction, by integrating expression data to check whether it could indeed occur, or by performing a RIP-qPCR assay in their system).

The second, most reliable method is obviously measuring several mRNA features in the system under study, focusing on a specific biological question, and then proceed by intersecting the obtained data to generate hypotheses stemming from the correlation of specific features. An intuitive example of this approach is in profiling the transcriptome and the translatome (the last through polysomal profiling, for instance) in various conditions (e.g., drug treatment vs. control) to identify which genes are subjected to translational control and the impact the treatment may have on translational efficiency (computed as the translatome vs. transcriptome ratio): this has already been done in a number of works (Genolet et al., 2011; Bates et al., 2012; Fu et al., 2012; Tebaldi et al., 2012; Courtes et al., 2013; Dudek et al., 2013; Willimott et al., 2013). A variation on this theme could include, in parallel, a miRNAs profiling in the system to correlate differences in their levels with differences in translational efficiency, generating candidate determinants of the latter changes (Clarke et al., 2012). Another example is the secondary structure and translational efficiency profiling of mRNAs in the system under study, aiming at the identification of structural patterns conferring translational advantages to the mRNAs containing them (Ding et al., 2014; Mao et al., 2014). Along the same line is coupling m6A methylation probing and RNA-seq measurements in the same system: this allows us to understand whether methylation alters mRNA level, stability and splicing patterns in the conditions under investigation (Dominissini et al., 2012; Zheng et al., 2013; Wang et al., 2014a).

The last integration method we describe is based on bridging the previous two approaches: combining a limited number of direct measurements performed in the system of interest with the wealth of data available in public databases such as the ones described above (even though these data may not be produced in the same model). One may thus investigate whether, for instance, an RBP or a miRNA is controlling a group of mRNAs, whether the gene set under analysis is enriched with a particular feature (e.g., a 3′UTR cis-element in the form of a secondary structure, methylated nucleotides, etc.) or match observed patterns for one feature type (e.g., presence of a secondary structure feature) with public data (e.g., presence of trans-factor binding sites) to deduce general rules (e.g., preference of a trans-factor for that given structural feature). While this method leads to hypotheses that need validation as they may not hold in the system of interest, it allows speeding up the investigation and reducing the hypotheses space, consequently lowering experimental uncertainty, time and cost. This approach has been enabled just recently, due to the availability of the databases discussed above. However, in the few published works adopting it, it is usually applied to the integration of data focused on a few specific mRNAs, which have been previously selected for their behavior as observed in the ongoing study (Mazza et al., 2013; Avery-Kiejda et al., 2014; Wang et al., 2014b). One exception is the recent work by Schueler and colleagues, in which protein contact sites obtained by a global PAR-CLIP on two cell lines are integrated with known RBP binding sites to infer differential protein occupancy patterns (Schueler et al., 2014).

Summing up, even though the approaches we have discussed are useful examples of data integration applied to the structure and the behavior of mRNAs, it is evident that these are still early and limited efforts. Indeed, as also testified by the small number of published works, there still is a significant lack of accepted practices and standard procedures which could render these approaches of effective routine usage. Having built a database focused on post-transcriptional regulation (Dassi et al., 2014), we realized that processed data, as submitted by the authors, vary widely in their processing level: if we take CLIPs datasets as an example, some datasets include the definition of sites bound by the studied RBP while others are limited to, for instance, the indication of T > C conversions (for PAR-CLIP); obviously this marked differences put additional burden on whoever wants to use multiple datasets, produced in different experiments, together, in order to generate new hypotheses. Furthermore, methods are often described in many ways, with different levels of detail, representing further obstacles in individuating steps needed to make these datasets truly comparable. A last general issue is the absence of a systematic way to evaluate data quality and robustness, considering for example the presence of replicates, the number of supporting reads and other parameters linked to specific techniques.

## The need for standardization

Given the outlined issues, we asked which steps could be taken to improve the exploitability and the integration potential of the RNA-centered high throughput data. We propose two simple, preliminary actions. The first is the enforced use of standard file formats with precisely defined fields, a relatively simple goal to achieve. The second is the enforced provision of a minimal set of information—enhancing dataset description, uniformity and allowing quality evaluations—at submission time (similarly to what was established and is currently enforced for microarrays with MIAME and related initiatives; Brazma et al., 2001; Rayner et al., 2006). This could be straightforwardly imposed by repositories commonly used for high-throughput datasets submission such as GEO (Barrett et al., 2013), ArrayExpress (Parkinson et al., 2005), and SRA (Wheeler et al., 2008).

Concerning the first requirement, we need to deal with two types of data: intervals (such as RBP and miRNA binding sites obtained through CLIPs) and per-nucleotide intensities (continuous values such as the ones produced by RNA methylation or secondary structure probing assays). Intervals are most often represented by means of the Browser Extensible Data (BED) format: its main advantage lies in the extreme simplicity of fields definition, which nevertheless allows a certain degree of detail, making it also feasible to represent several datasets in a single file (by for instance using the name field to distinguish the RBP/miRNA and possibly specifying methods and data source publication in the description field). Furthermore, BED files can be converted to bigBed (Kent et al., 2010), the associated binary indexed format that is efficient to process and use with genome browsers even for huge datasets. Concerning continuous values, they are most often stored by means of either a format similar in nature to BED, called bedGraph, or through another common option called Wiggle (Kent et al., 2010). Both formats are stripped down to the essential and are not really intended to allow mixing different datasets in the same file; the file header however leaves room for some description to be added; furthermore, both can be converted to the binary indexed bigWig format (Kent et al., 2010), similarly to what mentioned above for bigBed. Given the versatility and already widespread use of these two formats, coupled with the storage and display efficiency, we propose that they should be deemed as de facto standards and systematically required for new data submissions.

For the second requirement (minimal set of parameters describing a dataset), which information should be considered as essential for the data to be exploited at their full potential? First of all, in the case of CLIP datasets intervals representing binding sites should be provided, rather than including raw per-nucleotide data only. Many scientists would not or cannot go the extra mile to compute intervals out of per-nucleotide data by themselves, and would thus loose the opportunity to use them. Furthermore, methods employed for data analysis should be described, at least briefly, indicating how intervals or per-nucleotide intensities (e.g., in the case of secondary structure data) were computed from raw reads. Eventually, basic quality metrics such as the number of replicates and the read depth supporting a given interval/position, along with call significance *p*-values (where appropriate) should also be provided to let the users judge on the data robustness, eventually allowing the application of homogeneous stringency filters when integrating multiple datasets. We believe that this “information package” could be enough to describe the data under study to an extent that will eventually make going back to the raw data unnecessary: we therefore propose that these information should be required when submitting a dataset of this sort.

Pushing further on this proposal, we may also consider the need for a dedicated repository storing transcriptome-centric positional data. Similarly to what major journals ask for microarrays-containing works, submission to this repository could be a de facto requirement for publication and have an unique ID assigned, to which direct reference could be made in publications further easing data traceability. Using one of the currently available databases as a repository of this sort could also have the advantage of allowing us to display various datasets together, integrated in a transcript-oriented way, thus providing a first glimpse of the data along with the possibility to retrieve them. Of course, this collection of proposals, which goes along the lines of several other “reproducible research” initiatives, can become a reality only if the majority of scientists in the field agree and commit to sustain it by complying with these recommendations.

## Conclusion

The availability of techniques based on high-throughput sequencing is fostering the investigation of the biological behavior of transcriptomes with an unprecedented level of detail and a continuously increasing amount of available data types: the very nature of this technology effectively allows us to pinpoint the location of features responsible for known and unknown biochemical properties of mRNAs and non-coding RNAs which may ultimately influence mRNA translation. However, the integration of these datasets is still in its infancy, with only a few approaches and applications in the literature and a lot of room for improving and making these efforts much easier and useful. We think that this process could be eased by committing to the introduction of standardization measures involving file formats, minimal information to be provided for dataset description and, possibly, the setup of a dedicated data repository. The choice to advance a proposal limited to transcripts biological features is justified in our opinion by the momentum gained by studies in post-transcriptional regulation of gene expression, by the several RNA-seq-based techniques introduced in the last 2 years, and the exponential growth of datasets of this type being released. We therefore think that the effort needed to implement such proposal could be worthy and fruitful. While certainly requiring coordination between laboratories studying the topic, initiatives like OBO (Smith et al., 2007), MIAPE (Taylor et al., 2007), and BioBricks (Smolke, 2009) have shown that it is possible to implement and sustain a standardization effort aimed in our case at a better exploitation of high-throughput data. Given the pace at which these data are accumulating, we need for sure to urgently push their integrated exploitation to its fullest extent.

### Conflict of interest statement

The authors declare that the research was conducted in the absence of any commercial or financial relationships that could be construed as a potential conflict of interest.
